# Descriptive study of association between quality of care and empathy and burnout in primary care

**DOI:** 10.1186/s12910-017-0214-9

**Published:** 2017-09-26

**Authors:** Oriol Yuguero, Josep Ramon Marsal, Miquel Buti, Montserrat Esquerda, Jorge Soler-González

**Affiliations:** 1 Institut Universitari d’Investigació en Atenció Primària Jordi Gol (IDIAP Jordi Gol), Barcelona, Spain; 2 Faculty of Medicine, University of Lleida, Lleida, Spain; 3 Institut Català de la Salut, Lleida, Spain; 4 Unitat de Suport a la Recerca Lleida, Institut Universitari d’Investigació en Atenció Primària Jordi Gol (IDIAP Jordi Gol), Mataró, Spain; 5 Cardiovascular Epidemiological Unit, Vall Hebron Hospital, Barcelona, Spain; 6 Borja Institute of Bioethics, Barcelona, Spain; 7 Institute for Biomedical Research in Lleida Dr. Pifarré Foundation, IRBLLEIDA. Av. Alcalde Rovira Roure, 80, 25198 Lleida, Spain

**Keywords:** Primary care, Quality of care, Medical ethics, Doctor-patient relationship, Health economics

## Abstract

**Background:**

The doctor-patient relationship is a crucial aspect of primary-care practice Research on associations between quality of care provision and burnout and empathy in a primary care setting could improve this relationship.

**Methods:**

Cross-sectional study of family physicians (108) and nurses (112) of twenty-two primary care centers in the health district of Lleida, Spain.

Empathy and burnout were measured using the Jefferson Physician Empathy Scale and the Maslach Burnout Inventory, while quality of care delivery was evaluated using Quality Standard Indicator scores. JPSE and MBI results were grouped into low, medium, and high scores to analyze associations with QSI scores and sociodemographic variables.

**Results:**

The mean QSI score recorded for the family physicians and nurses was 665 (out of a total of 1000). Higher, albeit insignificant, QSI scores were observed for practitioners with high burnout. No differences were observed according to level of empathy (*p* > 0.05). The differences with respect to sex, age, and area of practice (urban vs rural center) were not significant. Practitioners with low empathy had higher QSI scores than those with high empathy (672.8 vs. 654.4) while those with high burnout had higher scores than those with low burnout (702 vs. 671).

**Conclusions:**

Burnout and empathy did not significantly influence quality of care delivery scores in 22 primary care centers. More studies, however, are needed to investigate the unexpected trend observed that suggests that physicians and nurses with higher levels of burnout provide higher quality care.

## Background

The doctor-patient relationship is a crucial aspect of primary-care practice [1]. The face of primary care, however, has changed considerably in recent years with continuous advances in technology and scientific knowledge and increasing patient access to information. Practitioners have had to adapt to these changes, while simultaneously coping with increasing workloads and diminishing resources, to ensure that the patient remains at the center of care. [2] The potential impact of these changes on practitioners has been studied from numerous angles in recent years and two distinct yet related concepts have received particular attention: burnout and empathy. [3, 4]

In Spain, the patient medical relationship has presented a similar evolution to that of the rest of Europe. The existence of a public system that guarantees universal access for all citizens, allows all the people of our country to have a reference family doctor who can consult without limitation, and who manages most health problems. Access to hospital care is reserved for serious pathology and unless emergencies must be performed by prescription of a family doctor. The increased technification of the health system has facilitated the connection between different levels of care, but the multitude of data available from the patients can limit the clinical interview and base the patient doctor relationship in an analysis of tests and results.

Empathy is the ability to understand another person’s feelings and thoughts and to relay this understanding back to the person [5]. Empathic engagement by health care practitioners has been associated with numerous benefits related to doctor-patient communication [6], patient satisfaction, [7] and adherence to treatment. [8] Burnout has also become increasing relevant as practitioners have been exposed to growing workloads and increasing social pressures. [9] Burnout syndrome, as defined by Maslach, has three dimensions: emotional exhaustion, (low) personal accomplishment, and depersonalization of the doctor-patient relationship. [10]

A recent study of primary care physicians by our group found a significant association between empathy and burnout, with more empathic physicians experiencing lower burnout rates. [11] A greater understanding of the links between burnout and empathy has led to the development of programs and strategies designed to strengthen resilience [12] and empathic engagement [13] as mechanisms for preventing burnout in primary care. [14] According to the recently published 2016 Medscape Lifestyle report, [15] almost 46% of physicians reported job burnout.

At the other end of the spectrum, health care authorities are increasingly concerned with ensuring that patients receive the best quality care possible. [16, 17] One means of evaluating quality of care is through tools that objectively assess how closely performance in everyday practice meets recognized standards of excellence. Numerous such initiatives exist in Europe [18] and most national health ministries and institutes have established indicators to measure quality of care and identify gaps and areas for improvement. [19] Considering the current pressure on public resources, these initiatives also focus on making the best possible use of available resources. Actions implemented by health care centers in Europe have also been analyzed to identify and assess the efficacy of measures designed to improve quality of care. [20] An important component of such programs is to secure the participation of users and patient associations alongside health care professionals and scientific societies. [21]

Quality of care and its implications for primary care practice is a growing area of research. [22] The results of a recent study of primary health care teams in Barcelona, Spain suggest that services could be made more efficient by using quality indicators as output measures [23]. This is supported by the results of numerous studies that have found that strategies aimed at improving quality of care result in more efficient health care services. [24]

The Catalan health care system is undergoing numerous transformations aimed at creating a patient-based system resilient to the pressures brought on the economic and financial crises of recent years. [25]

In 2007, the health care district of Lleida introduced a Quality Standard Indicator (QSI) system designed to objectively measure the quality of care provided by primary health care teams operating at different centers. [26] One of the aims of this tool is to feed the continuous improvement of care delivery through the engagement of both physicians and nurses.

The QSI was created with the support of primary care institutions and societies and the criteria on which it is based have varied only marginally since its implementation in 2007. [27] The QSI addresses 20 of the most common health problems in primary care and is based on information entered into the centralized computer system by physicians and nurses during their day’s work.

In our review of the literature, we found no studies that have specifically addressed the association between quality of care and burnout and empathy of health care professionals. The aim of this study was to investigate whether the quality of care provided by primary care physicians and nurses varied according to levels of burnout and empathy.

## Methods

### Participants and study design

We undertook a descriptive study of family physicians and nurses working in the health care district of Lleida, a Catalan province in the north of Spain. The district has 22 primary care centers that serve a population of approximately 366,000 people. All the physicians and nurses registered in the district (*n* = 418) were contacted by e-mail and asked to voluntarily complete an anonymous survey on burnout and empathy between May and July 2014. All responses were anonymized to guarantee confidentiality.

### Instruments and variables

#### Main outcome variable

##### Quality of care assessment

The QSI provides an overall score of 0 to 1000 points, with higher scores indicating better quality of care. The indicators used to measure each of the 20 problems were established and reviewed by experts in the corresponding areas. [28] Each indicator has a series of minimum results that must be achieved. Indicators for which these minimum results (equivalent to the 20th percentile) are not achieved are assigned a score of 0, while those for which maximum results (80th percentile) are achieved are assigned the maximum possible score for the corresponding indicator. In-between results are allocated a score of between 1% and 99%. There are no cutoffs to indicate achievement of a minimum level of quality. The indicator is the result of some parameters collected by professionals and that have scientific consensus to be used as quality monitor in multiple aspects. There are parts that evaluate screening activities, others of secondary prevention, of therapeutic compliance, etc. Some examples of these indicators are achievement of a good control of blood pressure, to make good use of tumor markers or to perform retinopathy screening in diabetic patients. The final score is the result of some indicators registered by professionals and others obtained automatically by the computer system. The system is simply based on scores, with higher scores indicating better quality. The data provided by the questionnaire respondents were linked to their corresponding QSI scores.

#### Secondary outcome measures

##### Measurement of burnout and empathy

Burnout was measured using the Spanish version of the 22-item Maslach Burnout Inventory (MBI), which has been formally validated [29] and used in previous studies in our setting. [30] This scale measures the three dimensions of burnout: depersonalization, personal accomplishment, and emotional exhaustion. Empathy was measured with the Spanish version of the Jefferson Physician Empathy Scale (JSPE) [31], which is also a widely used, validated scale, consisting of 20 items [32]. The JSPE is the most widely used scale for assessing empathy in health professionals. An scale that measures physician empathic orientation and behavior. The average empathy score is considered to lie around 125 points and we followed previous strategies of classifying empathy levels as high for mean scores plus 2 SDs and as low for mean scores minus 2 SDs.

The MBI and the JSPE are scored on a 7-point Likert-type scale, with higher scores indicating higher levels of burnout and empathy, respectively.

##### Evaluation of information recorded by physicians and nurses

A key component of the QSI system is the amount of information entered daily by physicians and nurses into the centralized computer system. We collected this information for each of the participants in the study to investigate associations between level of activity and quality of care delivery.

##### Other variables

The following sociodemographic data were recorded: profession (physician or nurse), age, sex, and place of practise (urban, defined as a health care center in the capital city, vs.rural).

### Data analysis

The initial analysis comprised a descriptive study of the qualitative variables and MBI and JSPE scores. The reliability of the instruments was checked by calculating the internal consistency (Cronbach α) of the two tools. The corresponding scores were 0.733 for the MBI and 0.748 for the JSPE.

The Chi-square and Kolmogorov-Smirnov-Lilliefors tests were used to check the distribution of data and select the most appropriate coefficient for the correlation analyses. The Pearson coefficient was used for normally distributed data and the Spearman coefficient for non-normally distributed data.

To analyze associations between sociodemographic variables, burnout, empathy, and QSI, JSPE and MBI scores were classified as low, medium, or high according to a previously described system [12]. All the results were presented with 95% confidence intervals.

Associations were compared by chi-square analyses. Results were stratified by age, sex, profession, and place of work. Means, percentages, and standard deviations were calculated in SPSS version 15.0.

## Results

Higher QSI scores were observed for practitioners with high burnout, although the differences with practitioners in the low and medium burnout groups were not significant. No differences were observed according to level of empathy. The survey was answered by 108 physicians and 112 nurses, which corresponds to a response rate of 52.6%.

Table 1 shows the results for the 220 respondents according to level of empathy, which was dichotomized into high empathy (*n* = 77) and low/medium empathy (*n* = 143). No significant differences were found for sex, age, or area of practice (urban vs rural). High empathy was significantly associated with low burnout, as previously reported [11].

**Table 1:** Description of sample according to level of empathy

Empathy	Medium-Low (*n* = 143)	High (*n* = 77)	Total (*n* = 220)	p	OR (CI 95%)
	n (%)	n (%)	n (%)		
Gender (Women)	107 (74,8%)	64 (83,1%)	171 (77,7%)	0,159	1,66 [0,82–3,35]
Age	48,74 (8,6%)	47,64 (8,5%)	48,35 (8,6%)	0,36	0,99 [0,95–1,02]
Place of Work (Rural)	88 (61,5%)	39 (50,6%)	127 (57,7%)	0,119	0,64 [0,37–1,12]
Profession (Doctor)	65 (45,5%)	43 (55,8%)	108 (49,1%)	0,141	1,52 [0,87–2,65]
BURNOUT				0,02	
LOW	76 (53,15%)	57 (74,03%)	133 (60,45%)		Ref 1
MEIDUM	60 (41,96%)	19 (24,68%)	79 (35,91%)		0,42 [0,23–0,78]
HIGH	7 (4,9%)	1 (1,3%)	8 (3,64%)		0,19 [0,02–1,59]
Emotional Exhaustion				0,209	
LOW	79 (55,24%)	48 (62,34%)	127 (57,73%)		Ref 1
MEDIUM	27 (18,88%)	15 (19,48%)	42 (19,09%)		0,91 [0,44–1,89]
HIGH	37 (25.87%)	14 (18,18%)	51 (23,18%)		0,62 [0,31–1,27]
Depersonalization				0,038	
LOW	82 (57.34%)	59 (76,62%)	141 (64,09%)		Ref 1
MEDIUM	42 (29.37%)	9 (11,69%)	51 (23,18%)		0,3 [0,13–0,66]
HIGH	19 (13.29%)	9 (11,69%)	28 (12,73%)		0,66 [0,28–1,56]
Personal Accomplishment				0,0001	
LOW	18 (12,59%)	3 (3,9%)	21 (9,55%)		Ref 1
MEDIUM	69 (48,25%)	17 (22,08%)	86 (39,09%)		1,48 [0,39–5,6]
HIGH	56 (39,16%)	57 (74,03%)	113 (51,36%)		6,11 [1,7–21,89]

Table 2 shows mean QSI scores according to empathy and burnout levels. No significant associations were found between QSI scores and either burnout or empathy.

**Table 2:** Quality standard index (QSI) scores according to empathy and burnout scores

		QSI scores	
		Mean (SD)	
EMPATHY	N		*p* = 0.190
Low	73	672.8 (101.1)	
Medium	70	669.1 (166.9)	
High	77	654.4 (113.3)	
BURNOUT			*p* = 0.153
Low	133	671.8 (139.1)	
Medium	79	649.9 (113.7)	
High	8	702 (75.7)	

The mean QSI score for the overall group was 665 points. Practitioners with low empathy scored higher than those with high empathy (672.8 vs. 654.4), while those with high burnout scored higher than those with low burnout (702 vs. 671.8).

**Fig. 1:**
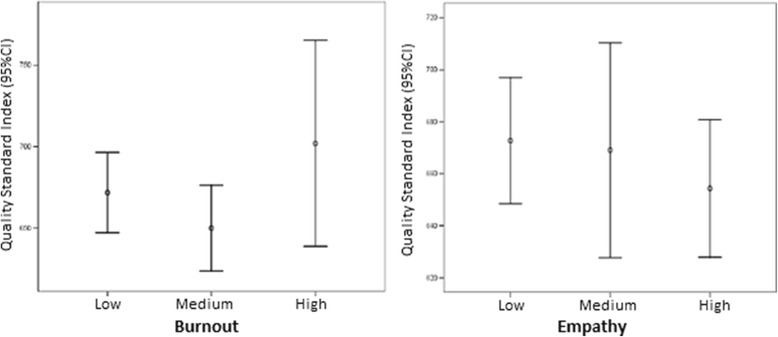
Boxplots of Quality Standard Index scores according to empathy and burnout scores

**Fig. 2:**
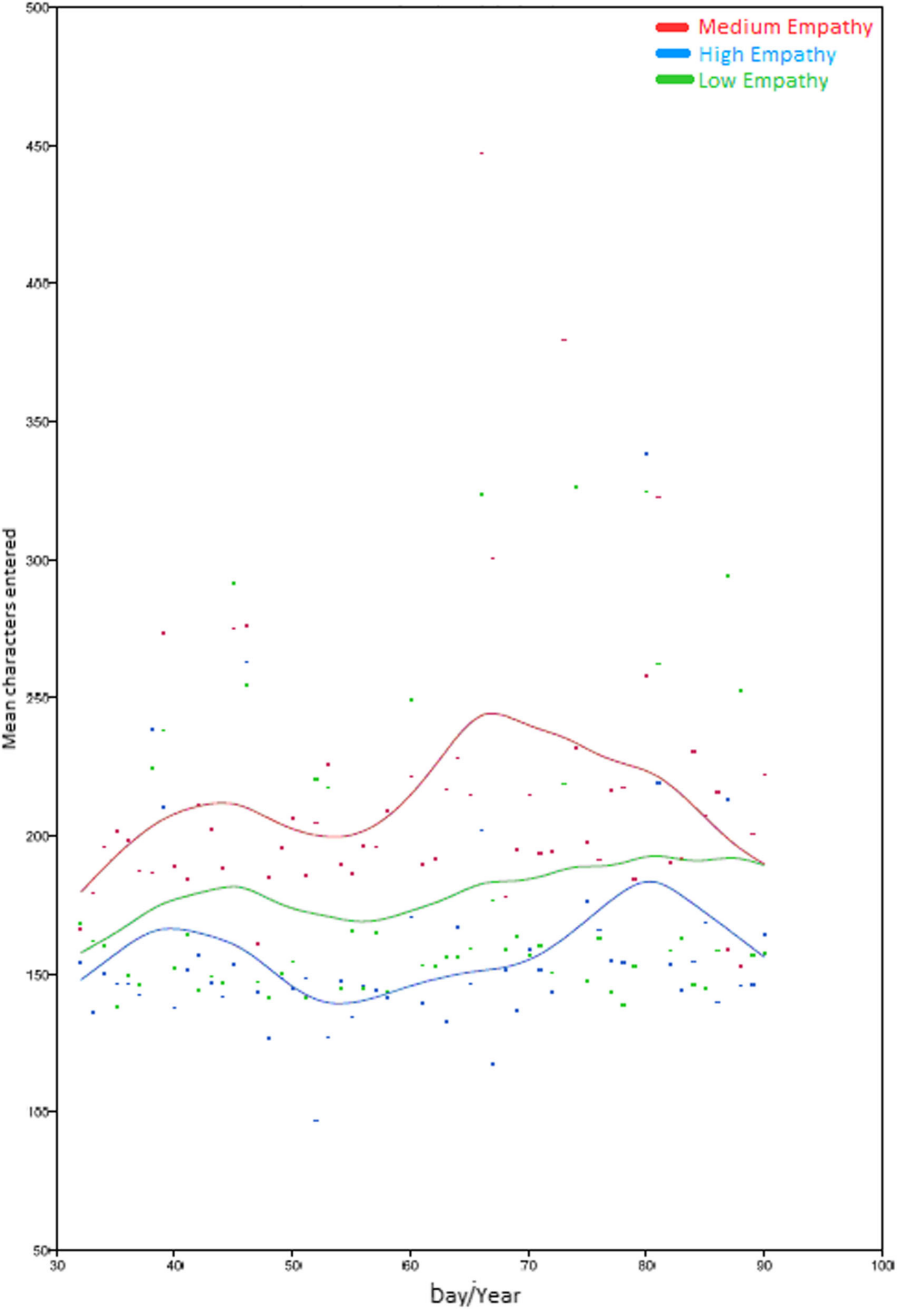
Graph showing sample of information entered into the centralized computer system by physicians and nurses according to empathy levels

As shown in Fig. 1, the relationship between QSI scores and burnout and empathy was not linear. This figure also shows that while the high burnout group contained the practitioners who scored highest on the QSI, it also contained a considerable number of practitioners with low scores. On analyzing the scores by level of empathy, those with moderate levels performed best. This result is consistent with the information shown in Fig. 2 below.

Figure 2 shows the results for the analysis of information entered in the centralized computer system according to level of empathy. The graph clearly shows that practitioners with the highest level of empathy typed in the least information.

## Discussion

Quality of care, as measured by QSI scores, was not associated with empathy or burnout in a sample of 220 family physicians and nurses.

Empathic engagement by health care professionals has been found to have a direct impact on patient health. [33] Empathic skills, and the ability to understand patients’ feelings and concerns, are basic to proper health care. With the empathic perspective, we can promote the patient’s autonomy, favoring his benefit and avoiding maleficence, all of them fundamental principles of ethics. A similar case might be made for burnout, as a recent study by our group, pending publication, [34] showed how blood pressure management and control in a primary care setting varied according to levels of practitioner empathy and burnout.

### *Quality and primary care*

Although potential mediators of quality of care have been analyzed from numerous angles, [35] to our knowledge, the present study is the first to link results based on a quality standard to aspects related to the person providing the service, i.e., empathy and burnout. There have, however, been studies of how clinical circumstances can cause burnout [36] as well as studies that have analyzed the quality of care delivered in practice.

The mean QSI score recorded for the family physicians and nurses in our study was 665. Although we used indicators corresponding to care delivered to over 300,000 patients, we found no statistical associations between quality of care and either empathy or burnout. Nonetheless, we did observe that practitioners with high burnout and moderate empathy performed better on the QSI. It is important to recall at this point that the QSI system analyzed is based on information recorded by members of the health care teams during their everyday activities. It is therefore dependent on the involvement of these professionals. On an annual report, the information recorded by professionals is evaluated. The indicator does not evaluate the amount of data, but the quality of the performance of professionals. Other parameters are obtained automatically, such as the performance of analytics or drugs that a professional can prescribe.

Although the differences were not significant, our results suggest that physicians and nurses with high burnout might be providing better care than their less burnt out peers. One would expect practitioners with higher levels of burnout to record less information in the system and score lower on the QSI. However, this was not the case. We are unable to compare our results with those of other authors due to a lack of similar studies but we do believe it would be interesting to analyze a larger sample to further investigate this trend. Our finding that physicians and nurses with high burnout performed best in terms of quality of care delivery is surprising, although of course this does not mean that they are necessarily better physicians or nurses than those with lower scores.

Our findings regarding the association between level of activity and empathy are important in this respect. The fact that practitioners with medium levels of empathy spent the most time entering patient information and scored highest on the QSI suggests that probably more empathic practitioners prior to spend more time talking to their patients than entering information and codes into the system. In other words, it would seem that moderately empathic physicians and nurses provide quality care while also displaying empathy.

This is why we believe that favoring the empathy of professionals, promoting communicative skills, mindfullness programs and educational projects from the earliest years of medical education has an intrinsic value in improving the physician-patient relationship (not just ethics) but also of quality of care.

### *Management implications*

The results of this study provide useful insights for health managers, particularly those involved in primary care, as they suggest that quality standards should perhaps not be based exclusively on information recorded by health care practitioners and that it might even be necessary to create more ambitious, realistic standards that do not depend on time spent recording information.

### *Limitations and future lines of research*

One of the main limitations of this study is the fact that empathy was measured by the JSPE. Although this is a widely validated tool, it is a self-reported questionnaire and as such the results are prone to social desirability bias, as empathy is a sensitive issue. Another limitation is that we were unable to group our results by health care center due to their anonymous nature. Our interpretation of data with respect to other findings was also limited by the scarcity of similar studies. In future studies, it would be interesting to conduct multiple logistic regression analyses to identify other factors that could influence QSI scores. Quality is a growing health care priority worldwide and it is therefore important to continue to develop tools and strategies capable of measuring performance and identifying areas for improvement. We believe that our study is a step forward in the investigation of the numerous factors and situations that influence care delivery in everyday practice.

## Conclusions

Burnout and empathy did not significantly influence quality of care delivery in a primary care setting, although we did detect an unexpected trend that suggests that family physicians and nurses with higher levels of burnout provide better quality care. More studies are needed to investigate this trend.
